# Comparative Hydrodynamic Study on Non-Aqueous Soluble Archaeological Wood Consolidants: Butvar B-98 and PDMS-OH Siloxanes

**DOI:** 10.3390/molecules27072133

**Published:** 2022-03-25

**Authors:** Michelle Cutajar, Robert A. Stockman, Susan Braovac, Calin Constantin Steindal, Angeliki Zisi, Stephen E. Harding

**Affiliations:** 1 National Centre for Macromolecular Hydrodynamics (NCMH), School of Biosciences, University of Nottingham, Sutton Bonington LE12 5RD, UK; 2 School of Chemistry, University Park Campus, University of Nottingham, Nottingham NG7 2RD, UK; robert.stockman@nottingham.ac.uk; 3 Museum of Cultural History, University of Oslo, Kabelgata 34, 0580 Oslo, Norway; s.d.braovac@khm.uio.no (S.B.); c.c.steindal@khm.uio.no (C.C.S.); angeliki.zisi@khm.uio.no (A.Z.)

**Keywords:** analytical ultracentrifugation, Butvar B-98, PDMS-OH, Oseberg artefacts

## Abstract

Butvar B-98 and PDMS-OH both have a demonstrable ability as consolidants for archaeological wood. This makes them both potential treatment options for the Oseberg collection, which is one of the most important archaeological finds from the Viking era. Both Butvar B-98 and PDMS-OH are soluble in organic solvents, offering a useful alternative to aqueous-based consolidants. Extensive characterisation studies were carried out on both of these polymers, with the use of analytical ultracentrifugation and viscometry, for the benefit of conservators wanting to know more about the physical properties of these materials. Short column sedimentation equilibrium analysis using SEDFIT-MSTAR revealed a weight-average molar mass (weight-average molecular weight) *M*_w_ of (54.0 ± 1.5) kDa (kg · mol^−1^) for Butvar B-98, while four samples of PDMS-OH siloxanes (each with a different molar mass) had an *M*_w_ of (52.5 ± 3.0) kDa, (38.8 ± 1.5) kDa, (6.2 ± 0.7) kDa and (1.6 ± 0.1) kDa. Sedimentation velocity confirmed that all polymers were heterogeneous, with a wide range of molar masses. All molecular species showed considerable conformational asymmetry from measurements of intrinsic viscosity, which would facilitate networking interactions as consolidants. It is anticipated that the accumulated data on these two consolidants will enable conservators to make a more informed decision when it comes to choosing which treatment to administer to archaeological artefacts.

## 1. Introduction

Archaeological artefacts are unique time-travelling devices, transporting information from the past to the present. These artefacts not only provide us with a historical record but also with a source of cultural identity. It is therefore important that they are preserved for future generations. The Oseberg collection, discovered and excavated in 1903 and 1904 respectively [1], is comprised of highly valuable wooden artefacts and there is currently a race against time to develop new methodologies to reduce their active degradation and increase their strength. This unique assembly of archaeological artefacts was treated with hot alum salt solutions (KAl(SO_4_)_2_·12H_2_O and NH_4_Al(SO_4_)_2_·12H_2_O) between 1905 and ca. 1912 [2,3].

While this successfully provided support for the weakened structure of the waterlogged wood, it also inadvertently led to the formation of a very acidic environment in the wood (pH ca. 1–2.5) and the highly degraded state that the artefacts are presently in [4,5]. The severe decay of the Oseberg artefacts is such that many are only held together by the alum present in their wooden structure. Additionally, many objects have been reconstructed in ways that are not possible to undo without causing further damage. The objects with the highest degree of degradation and those which have been highly reconstructed cannot be re-treated with polymers in aqueous solutions. This is because the alum remaining in the artefacts may dissolve in water and consequently diffuse from the wooden structure, leading to total disintegration. As a result, it is deemed preferable to treat the most deteriorated and highly reconstructed artefacts with non-aqueous methods.

In this study, we carried out the extensive characterisation of two commercially available polymers representing such non-aqueous treatments, Butvar B-98 and hydroxy-terminated polydimethylsiloxane (PDMS-OH). These are currently being considered as candidates for the retreatment of part of the Oseberg wooden collection of artefacts. The performance of a wood consolidant depends not only on its chemical composition but also on its physical properties, notably molecular weight and molecular weight distribution and conformation, both of which affect the property to penetrate through the wood and interact/ form stable networks with wood components. In so doing, we will extend/reinforce the information already available for these materials, thereby assisting an archaeological wood conservator in his/her choice of the appropriate material.

This level of characterisation, in terms of size distribution, molar mass, and conformation of these two consolidants have never been carried out according to our knowledge, and we are anticipating that it will prove to be very useful when it comes to deciding which consolidants to choose for retreatment. Moreover, it will also be helpful to other future conservation projects.

**Figure 1:**
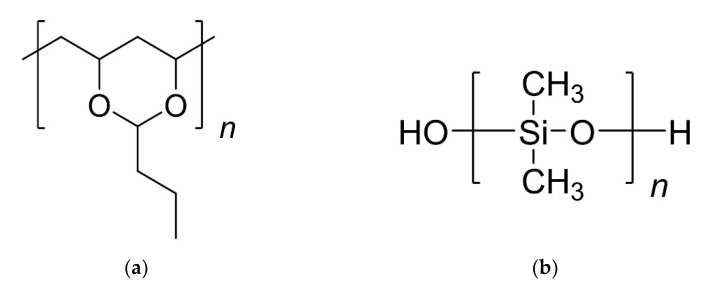
Structures of (**a**) Butvar B-98 and (**b**) PDMS-OH siloxanes.

Butvar B-98 is a polyvinyl butyral-based resin produced by the reaction of polyvinyl alcohol with butyraldehyde [6,7] (Figure 1a). It has already been used as a conservation treatment for archaeological artefacts, such as wooden objects excavated from tombs dating back to the 8th century BCE in Gordion, Turkey [6]: samples from these artefacts were consolidated with Butvar B-98 diluted in a solution of toluene and ethanol. After drying, it was shown that the wood had little or no shrinkage and minimal colour change. Spirydowicz et al. [6] reported that treating samples with 10% Butvar B-98 resulted in stabilisation without any significant micromorphological changes to the structure of the wood. Using scanning electron microscopy, it was confirmed that while the Butvar B-98 filled some of the cell lumina, it commonly coated their surfaces/cell walls.

Silicone-based polymers such as PDMS-OH (Figure 1b) possess a number of desirable properties such as hydrophobicity, high chemical, and temperature resistance, and minimal flammability [8]. These polymers form a macromolecular network in the wood via crosslinking, achieved by condensing the silanol groups to form siloxane bonds [8]. Kavvouras et al. [8] investigated the use of PDMS-OH together with a catalyst and a crosslinker, for conserving archaeological waterlogged wood from the Neolithic period in Greece. The process resulted in the formation of a three-dimensional polysiloxane network which effectively protected against the shrinking of the wood cells, whilst retaining the appearance of natural wood.

The characterisation described in the present study was primarily carried out by analytical ultracentrifugation, an absolute and matrix-free technique commonly used in the study of macromolecules [9]. This method has been recently described for the study of potential wood consolidants [10,11,12,13,14]. It is particularly attractive when compared to the more classical method of gel-permeation chromatography (GPC) since apart from the determination of particle sizes and shapes, it can also be used for the quantitative analysis of their interactions in solutions [9]. Moreover, unlike GPC, it can directly give molecular weights without the use of calibration standards of known molecular weight and a shape identical to that of the polymer being characterised. This eliminates the uncertainty that a method such as GPC generates when the polymer being tested does not have the same conformation as the standards employed.

There are two principle techniques that are carried out by analytical ultracentrifugation: a sedimentation velocity study and a sedimentation equilibrium experiment. Sedimentation velocity is a powerful matrix-free method that is used to gain information about the size distribution in terms of the sedimentation coefficient *s* (in Svedberg units, S), where s is the sedimentation rate per unit centrifugal field. It can also provide information on the shape of the macromolecule, as well as its interactions in solution. Since it generally works at relatively high rotor speeds, a high centrifugal force is developed which tend to dominate back diffusion effects. Although it is highly resolving, the *s* depends not only on the size but also on the shape of the molecule since extended molecules sediment slower. In contrast, sedimentation equilibrium is run at lower speeds; during such an experiment, the sedimentation and diffusion forces come to an equilibrium, and this provides direct information on the molar mass of the macromolecule, as there are no shape or friction effects, and without the need for standards. It also requires much shorter columns, minimising the effects of solvent compressibility issues. We also investigated the viscosity/conformational (shape) using a rolling ball viscometer [14] (with data analysed using the programme ELLIPS1) [15,16]. The primary solvent we used was isopropanol because the studied polymers are soluble in this; there is a sufficient density and viscosity difference between polymer and solvent, and it also has lower compressibility compared to many other organic solvents [17,18]. 

**Figure 2:**
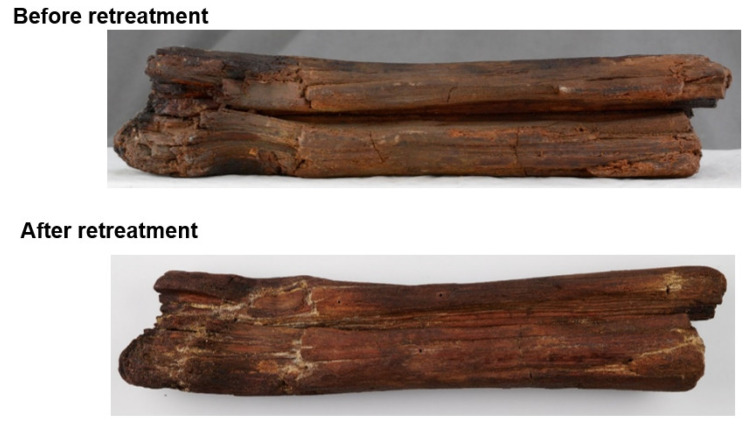
Wood fragment from the Oseberg collection before and after retreatment by injection with siloxane PS36000 in turpentine at a concentration of ~5% (50 g/L). The fragment had been originally treated with alum and linseed oil shortly after excavation in 1904.

We also investigated the viscosity and shape behaviour of the siloxanes in turpentine as a second solvent. Turpentine (Figure 2) was chosen as an additional solvent for investigation as siloxanes are soluble in it and it is also a natural or “green” solvent consisting of terpenes obtained from the wood of pine trees by steam distillation. This was not possible for the ultracentrifuge studies due to the insufficient density difference of the siloxanes with the solvent, nor for Butvar B-98 due to its insolubility in turpentine.

## 2. Results

### 2.1. Butvar B-98

#### 2.1.1. Sedimentation Velocity in the Analytical Ultracentrifuge

**Figure 3:**
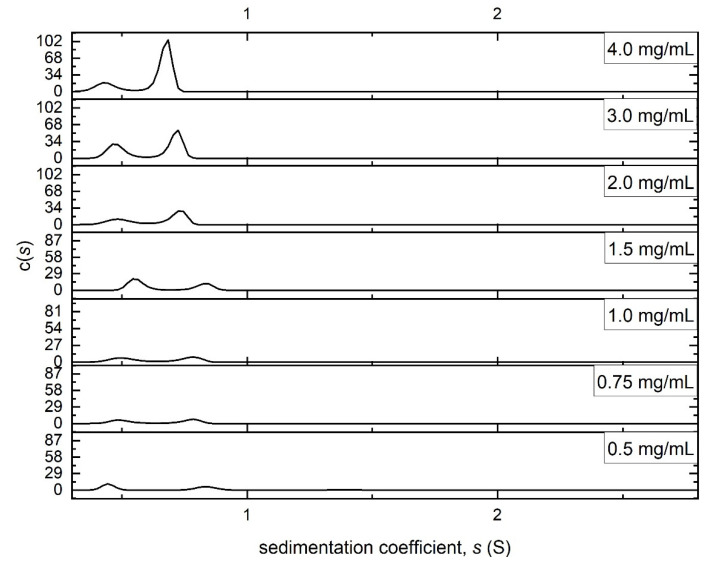
Sedimentation coefficient distributions of Butvar B-98 at different loading concentrations in isopropanol. Rotor speed = 49,000 rpm, temperature = 20.0 °C.

**Figure 4:**
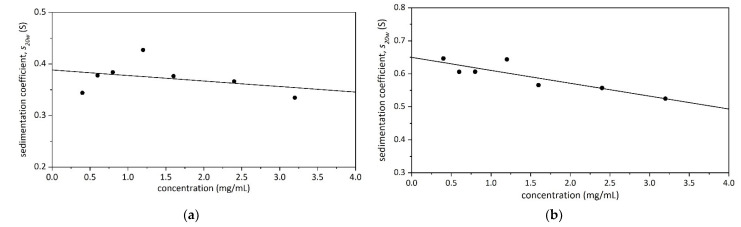
Dependence of *s*_20,w_ on sedimenting concentration (corrected for radial dilution) for Butvar B-98. (**a**) Slower component *s*^o^_20,w_ = (0.40 ± 0.02) S; (**b**) Faster component *s*^o^_20,w_ (0.65 ± 0.02) S.

Figure 3 shows the sedimentation coefficient distribution c(*s*) [19] vs. *s* of Butvar B-98 in isopropanol, analysed using the algorithm SEDFIT [20]. The analysis revealed material of a low sedimentation coefficient (<1 S) and close to the lowest limit of the technique (~0.4 S). No evidence of aggregation products was seen at higher sedimentation coefficient values. Low sedimentation coefficients are a feature of either low molar masses, extended conformations, or a combination of both. Two peaks are distinguishable at ~0.4–0.5 S and ~ 0.7–0.8 S. SEDFIT also enables the normalisation of the sedimentation coefficient to standard conditions (density and viscosity of water at 20.0 °C) for comparative purposes [21]. The normalised sedimentation coefficients (*s*_20,w_) for the two peaks were then plotted against concentration (Figure 4). It has to be stressed that these are on the lowest limit of sedimentation coefficient measurement.

#### 2.1.2. Sedimentation Equilibrium in the Analytical Ultracentrifuge

Sedimentation equilibrium experiments were then performed in order to determine weight-average molar masses. The algorithm SEDFIT-MSTAR [22] was used to evaluate the apparent weight-average molar mass *M*_w,app_ at cell loading concentrations ranging from 0.5 mg/mL (the minimum concentration required to give an adequate concentration distribution at sedimentation equilibrium) to 5.0 mg/mL (Supplementary Table S1).

Values are apparent at finite concentration because of the effects of thermodynamic non-ideality through molecular excluded volume effects [21], which leads to an underestimate of the true molar mass value. Depending on the size of the molecule, at sufficiently low concentrations *M*_w,app_ can be considered to be approximately equal to *M*_w_, the thermodynamically ideal value. Alternatively, measurements of *M*_w,app_ can be made at a series of concentrations, *c*, and extrapolated back to *c* = 0 where non-ideality effects are eliminated and hence, *M*_w,app_ = *M*_w_. The *M*_w,app_ values themselves are obtained from SEDFIT-MSTAR using either (i) the M* function, or (ii) the hinge method (see Schuck et al. [22]).

**Figure 5:**
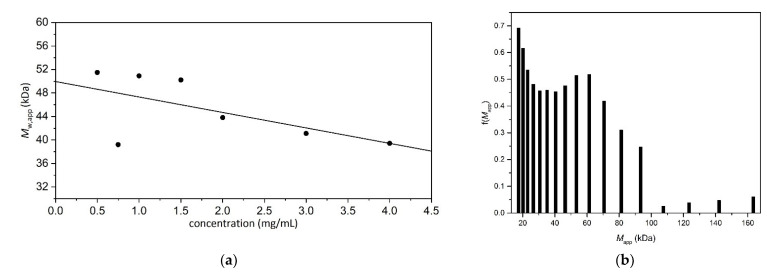
Sedimentation equilibrium of Butvar B-98 in isopropanol. Rotor speed 22,000 rpm. Temperature = 20.0 °C. (**a**) Dependence of the apparent *M*_w,app_ on concentration, with an extrapolation to obtain the thermodynamically ideal *M*_w_ of Butvar B-98 = (54.0 ± 1.5) kDa. The data was analysed with SEDFIT-MSTAR using the M* method. (**b**) Estimation using MULTISIG of the molar mass distribution f(*M*) vs. *M*_app_ at a loading concentration of 4.0 mg/mL. A broad distribution is seen consistent with sedimentation velocity (see Figure 3).

Figure 5a shows an extrapolation to zero concentration to eliminate non-ideality effects to yield an “ideal” weight-average molar mass *M*_w_ = (54.0 ± 1.5) kDa for Butvar B-98. SEDFIT-MSTAR also provides an estimate for the (apparent) z-average molar mass, *M*_z,app_, and hence the apparent polydispersity *Đ* = *M*_z,app_/*M*_w,app_.

The sedimentation equilibrium data for the 4.0 mg/mL loading concentration were additionally analysed with the MULTISIG algorithm. This programme utilises a 17-component system with 20 iterations to provide a distribution of the (apparent) molar masses in a polymer system [23]. Figure 5b reveals a broad distribution ranging from 17.5 to 163.4 kDa, with components peaking at 17.5 and 61.4 kDa for Butvar B-98. Both the weighted average whole distribution value *M*_w_ = (54.0 ± 1.5) kDa and the f(*M*) vs. *M*_app_ distribution are consistent with values previously quoted for Butvar B-98, namely 40–70 kDa [24].

### 2.2. Siloxanes: PDMS-OH

Four different preparations of PDMS-OH were studied across a range of molar masses assigned by the manufacturer based on size exclusion chromatography as 36,000 Da and 18,000 Da (both relative to polystyrene standards), and 4200 Da and ~550 Da: these samples are hitherto named as PS36000, PS18000, PS4200, and PS550.

#### 2.2.1. Sedimentation Velocity in the Analytical Ultracentrifuge

**Figure 6:**
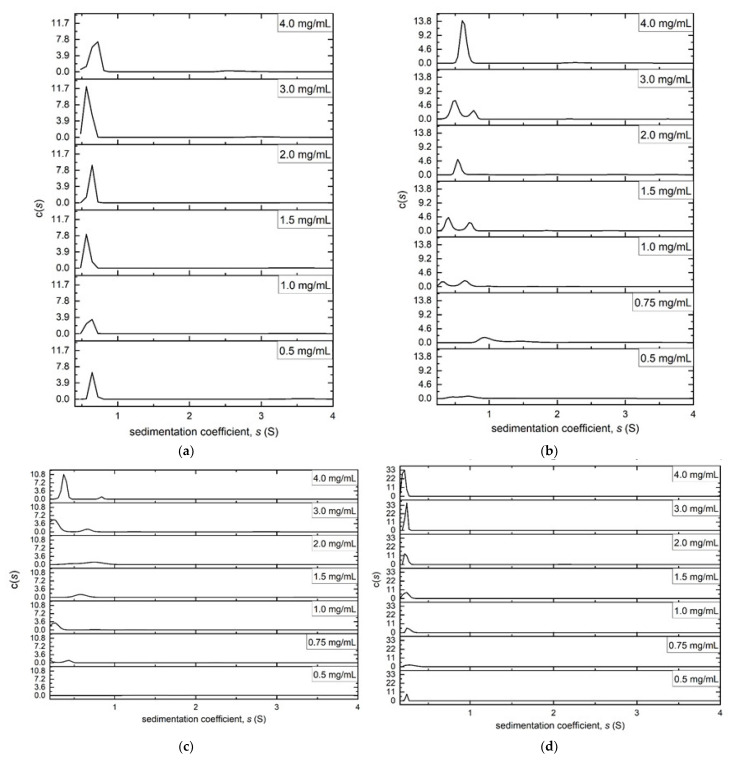
Sedimentation coefficient distributions of the PDMS-OH series at different loading concentrations in isopropanol. Rotor speed = 49,000 rpm, temperature = 20.0 °C (**a**) PS36000, (**b**) PS18000, (**c**) PS4200, (**d**) PS550.

The PDMS-OH series was solubilised in isopropanol. A rotational speed of 49,000 rpm at a temperature of 20.0 °C was used for all samples: Figure 6a–d compares the sedimentation coefficient distributions obtained from these experiments for the four samples across the range of concentrations. No evidence of high molar mass aggregation products was evident across the range. PS36000 (Figure 6a) had a single peak with a sedimentation coefficient value *s* of ~0.7 S. In contrast, PS18000 (Figure 6b) revealed two components. As the concentration increased, these two peaks showed a tendency to merge, possibly due to the Johnston—Ogston [25] effect where the faster moving component was slowed down by having to move through a solution of the slower moving component. PS4200 (Figure 6c) had very similar results to PS18000, especially at higher concentrations, where two primary components were observed. For PS550 (Figure 6d) the sedimentation coefficient was too small to be reliably detected, but the distribution plot did not show the presence of higher molar mass aggregates. When an attempt was made to calculate the *s*_20,w_ of the PDMS-OH samples, that is, the sedimentation coefficient normalised to the standard solvent conditions of the density and viscosity of water at 20.0 °C, it was discovered that all values came out negative. This would indicate that, had these molecules been soluble in water they would have moved in the opposite direction to the ultracentrifugal field—i.e., *flotation velocity*—somewhat similar to what we had previously observed for another polymer, ‘CoPo9′, a block copolymer made of poly(ethylene oxide), poly(isoprene) and poly(ethylene oxide)—which is soluble as unimers in chloroform but forms micelles in water [26].

#### 2.2.2. Sedimentation Equilibrium in the Analytical Ultracentrifuge

**Figure 7:**
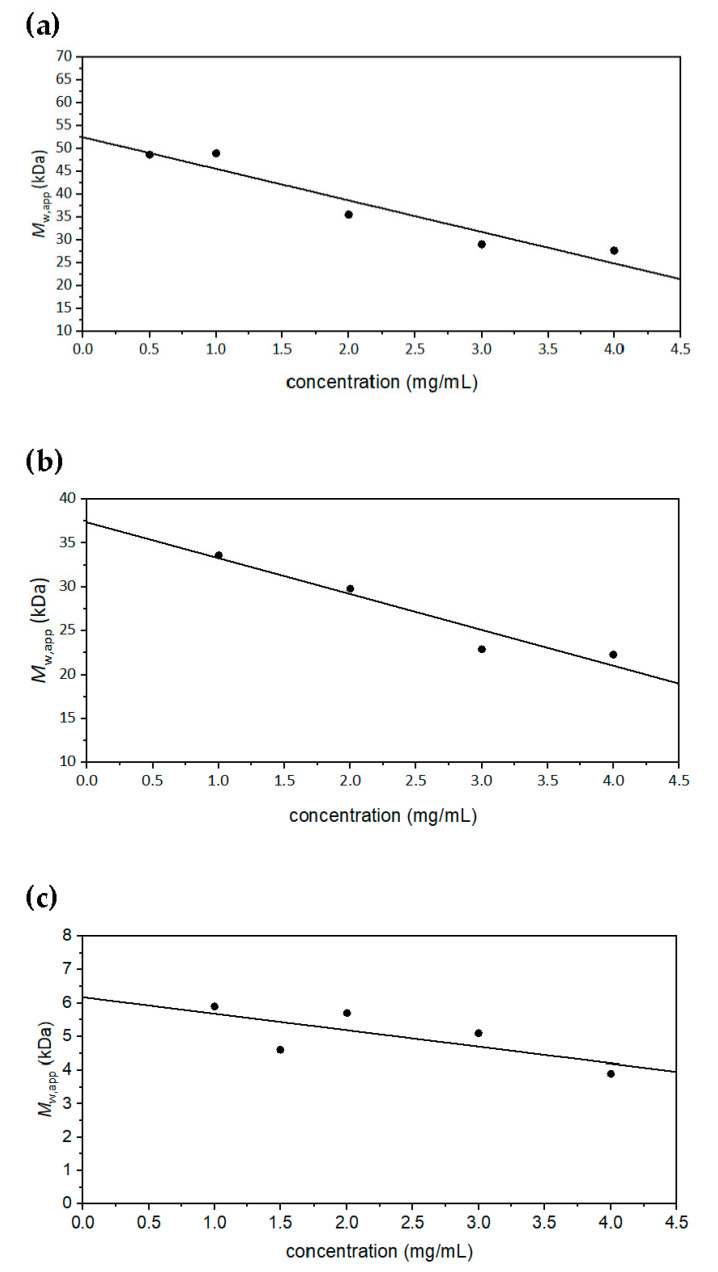
Dependence of apparent *M*_w,app_ from sedimentation equilibrum using SEDFIT-MSTAR on concentration, with an extrapolation to obtain the thermodynamically ideal *M*_w,app_ of the PDMS-OH siloxanes in isopropanol. (**a**) PS36000, rotor speed = 22,000 rpm, temperature = 20.0 °C. The value extrapolated to zero concentration was calculated to be *M*_w_ = (52.5 ± 3.0) kDa. (**b**) PS18000 in isopropanol, rotor speed = 30,000 rpm. *M*_w_ = (37.4 ± 2.3) kDa. (**c**) PS4200 in isopropanol, rotor speed = 49,500 rpm. *M*_w_ = (6.2 ± 0.7) kDa.

Sedimentation equilibrium experiments were performed on all samples, using appropriate rotational speeds according to their approximate sizes. (Supplementary Tables S2–S5). The thermodynamically ideal whole distribution weight average *M*_w_ values were obtained, as with Butvar B-98, by plotting the apparent *M*_w,app_ values against concentration and extrapolating to zero concentration (Figure 7). These weight-average molar mass values proved to be a little higher than the relative molar mass estimates *M*_r_ (relative to polystyrene standards) provided by the manufacturer (Table 1): this may be due to the different conformations of the siloxanes to the polystyrene standards in the solvent used. Because of the small size of PS550, measurable fringe increments were only possible at the higher concentrations, therefore *M*_w_ is obtained by taking the average of the 3.0 mg/mL and 4.0 mg/mL measurements (Table S5). Approximating *M*_w_ ~ *M*_w,app_ is justified because its exclusion volume/ non-ideality effects will be minimal [21].

**Figure 8:**
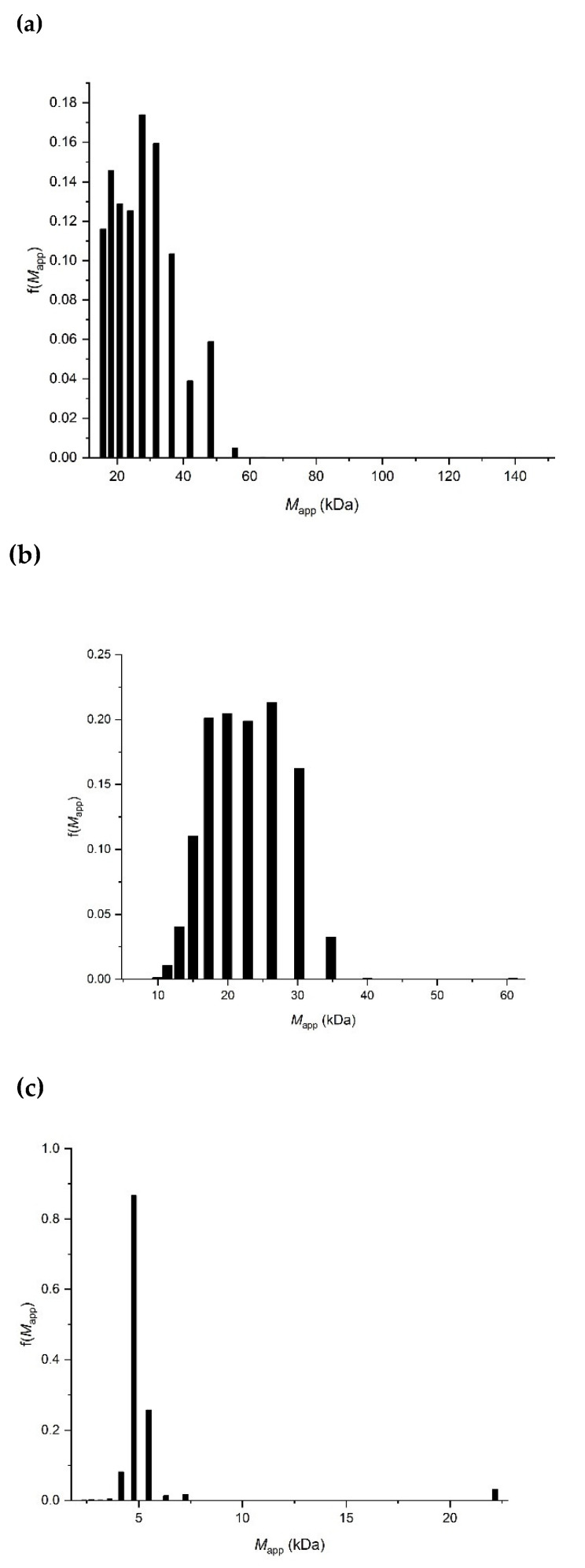
MULTISIG analysis of the molar mass distribution f(*M*) vs. *M* of PDMS-OH siloxanes in isopropanol at a concentration of 4.0 mg/mL and temperature = 20.0 °C. (**a**) PS36000; (**b**) PS18000; (**c**) PS4200.

Figure 8a–c shows the corresponding molar masses at a loading concentration of 4.0 mg/mL from the MULTISIG [23] analyses of the equilibrium data (the PS550 was not possible for this type of analysis due to its small size). The polymers appear to be fairly heterogeneous, with each having a wide range of molar mass.

### 2.3. Intrinsic Viscosity [η]

Intrinsic viscosity [21,27] measurements on Butvar B-98 and PDMS-OH were carried out with a rolling ball viscometer at 10.0 °C in isopropanol. The PDMS-OH samples were additionally measured in turpentine. The concentration of the polymer solutions that were used for these experiments was 6.0 mg/mL to give a sufficient flow time difference to that of the solvent. The [*η*] values (Table 2) were evaluated using the Solomon–Ciuta equation:

(1) $$\left[\eta \right]=\frac{1}{c}{\left(2\left(\eta_{sp}\right)-2ln\left(\eta_{r}\right)\right)}^{\frac{1}{2}}\;\;\;\;\;\;$$

where *η_r_* is the relative viscosity (ratio of the viscosity of the solution to that of the solvent) and *η_sp_*, the specific viscosity = *η_r_* − 1.

### 2.4. Conformation Analyses

**Figure 9:**
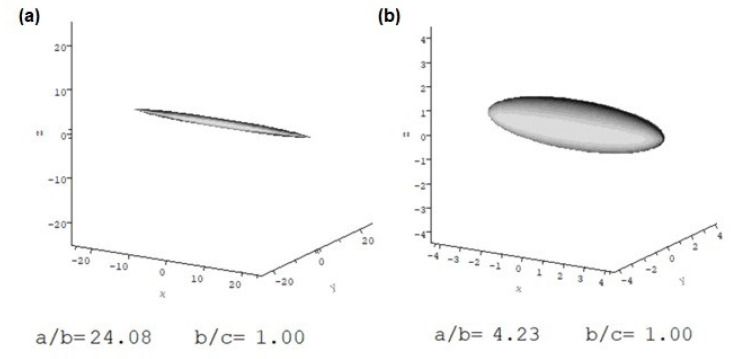
Conformation in isopropanol (equivalent hydrodynamic ellipsoids) for ν at $\left(v_{s}/\bar{v}\right)$ = 1.2 using the programme ELLIPS1 of (**a**) Butvar B-98 in isopropanol; (**b**) PS36000 siloxane in isopropanol. ELLIPS1 representations of PS18000, PS4200, and PS550, as well as polymers in turpentine, are shown in Supplementary Figure S1.

The programme ELLIPS1 [15,16] was used to provide an estimate of polymer conformation or asymmetry in terms of the axial ratio of the equivalent hydrodynamic ellipsoid (prolate) of the polymers using the viscosity increment (ν) shape factor. These models do not take into account flexibility effects, but nonetheless provide a useful relative comparison of asymmetry. A similar exercise has been reported recently for a potential consolidant derived from terpenes [14]. ν is related to [*η*] by the relation [28,29,30]:

ν = [*η*]/*v_s_* (2)

where *v_s_* is the swollen specific volume. Table 3 and Table 4 show these shape functions in terms of their aspect (axial) ratios (*a/b*) for ellipsoids of revolution [16] using a plausible range of solvent associations. Figure 9 (and Supplementary Materials) gives some examples.

#### Mark–Houwink–Kuhn–Sakurada (MHKS) Plots

**Figure 10:**
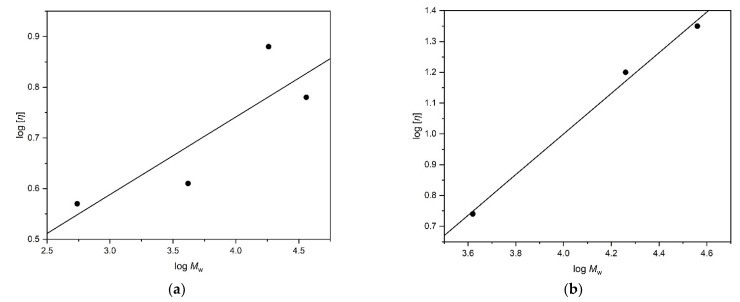
Mark–Houwink–Kuhn–Sakurada plots of the siloxanes in (**a**) isopropanol (a ~ 0.15) and (**b**) turpentine (a ~ 0.66).

From Table 1 and Table 2 it is possible to construct Mark–Houwink–Kuhn–Saurada plots of log [*η*] vs. log *M*_w_ (Figure 10) for the siloxanes to yield the MHKS *‘a’* coefficient from the slope where

[*η*] = *K*_η_*M*_w_*^a^* (3)

and *K*_η_ is a constant. MHKS *a* values range from ~0 for a spherical/globular particle, 0.5–0.8 for a flexible-extended coil, and >1.2 for a rod (see for example Harding [27]). In isopropanol, the slope “*a*”~(0.15 ± 0.07) is consistent with a spherical/globular approximation so the application of the ellipsoid models is justified. In turpentine “*a*” = ~(0.66 ± 0.05), which is approaching an extended coil, again consistent with the aspect ratios shown in Table 4.

## 3. Discussion

In this study, the characterisation of the consolidants Butvar B-98 and four different PDMS-OH series were successfully carried out, primarily with the use of analytical ultracentrifugation. The sedimentation equilibrium studies revealed that Butvar B-98 had a *M*_w_ of ~54.0 kDa, while the different samples of PDMS-OH had an *M*_w_ of ~52.5 kDa, 38.8 kDa, 6.2 kDa, and 1.6 kDa. The experimental *M*_w_ value of Butvar B-98 was shown to be consistent with the data provided by the manufacturer based on size exclusion chromatography coupled to a low angle light scattering detector, which like sedimentation equilibrium, is an absolute method not requiring assumptions concerning polymer conformation. Our absolute values for the weight-average molar masses for the siloxanes may provide a more accurate measure of molar mass to the previously available relative molar mass values, which were relative to polystyrene standards. The differences are probably due to the different conformations of the siloxanes and the standards. Sedimentation velocity, reinforced by the MULTISIG analysis of sedimentation equilibrium solute distributions, demonstrated that all polymers were heterogeneous with a broad range of molar masses. This may affect how efficiently these polymers can penetrate and consolidate archaeological wood.

**Figure 11:**
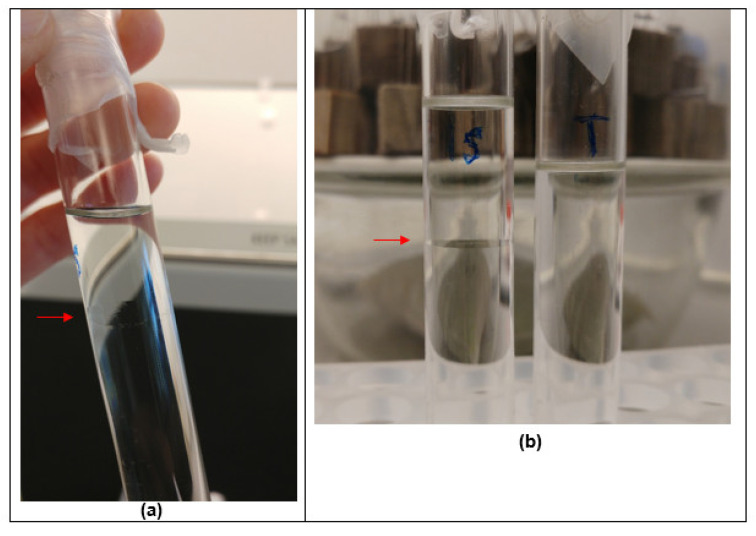
(**a**) PS36000 in isopropanol, showing phase separation of components as indicated by the red arrow. (**b**) = A comparison of PS36000 in isopropanol (**left**) and turpentine (**right**) after one month.

Conformational analyses in isopropanol confirmed that the polymers, in general, possessed an elongated shape. This was especially true for Butvar B-98, with the viscosity increment ν indicating an axial ratio of ~25 (allowing for a plausible solvation range). The different samples of PDMS-OH had much less extended conformations, with aspect ratios in the range from 2–5. In turpentine the two largest samples appeared to be more extended, which is also reflected in their increase in intrinsic viscosity (Table 2). This difference may relate to observations on the use of PS36000 in both turpentine and isopropanol to treat degraded archaeological wood [31]. Figure 11a shows separation between the PS36000 and solvent almost immediately after mixing it with isopropanol. In contrast, such a separation is absent in the mix of the polymer with turpentine, even after one month (Figure 11b). The more extended conformation of PS36000 in turpentine is most likely due to a greater solvent interaction with turpentine.

Nonetheless, three different concentrations of the polymer in the two solvents, 30%, 50%, and 70%, were used to immerse a number of archaeological wood test specimens for a period of two weeks during experimental work in the framework of the Saving Oseberg project [31]. Due to the experimental set-up, test specimens were layered in the immersion bath. When the weight percentage gain of the wood test specimens was calculated for all concentrations and solution types, for those stacked at the top of the isopropanol/polymer bath, all had consistently gained the least weight. This was in contrast to the test specimens immersed in the turpentine/polymer bath. In this case, there was no relationship observed between the weight percentage gain and the positioning of a test specimen in the immersion bath. Essentially, the wood test specimens sitting on the top part of the polymer/isopropanol bath were exposed to a lower polymer concentration than aimed for, due to the separation of the two components.

The practical meaning of this for a conservator is that, for instances involving application of the large molar mass siloxanes such as PDMS-OH PS36000 as a consolidant in wood via immersion, and considering the prolonged periods of immersion time, the preferable solvent to work with would be turpentine to ensure the desired amount of consolidant uptake by the wood—and this choice is reinforced by it’s natural or “green” origins from pinewood. However, solutions of PS36000 in isopropanol could be recommended for applications on wood via injection—as long as the mixture is gently shaken before injection—since short application times are involved. Moreover, since a consolidant application via injection brings conservators in close contact with a wooden object, the choice of a solvent that is less harmful to human health, isopropanol, is better than turpentine in this case. Lengthy evaporation rates (months to years) of turpentine may also be considered problematic.

## 4. Concluding Remarks

Butvar B-98 and PDMS-OH both possess the common advantage of being soluble in organic solvents. This is particularly important for the Oseberg artefacts, as avoiding aqueous treatment will help in preventing further possible damage during reconservation. The work described here has successfully expanded the knowledge base of these two polymers, which will hopefully result in a higher confidence level when it comes to deciding which consolidants to use in the retreatment of the Oseberg finds, as it will certainly do for other archaeological artefacts.

## 5. Materials and Methods

### 5.1. Materials

All reagents and solvents were purchased from a chemical supplier (Acros Organics Ltd., Geel, Belgium; Alfa Aesar Ltd., Haverhill, MA, USA; Merck Ltd., Darmstadt, Germany; or Fisher Scientific Ltd., Loughborough, UK) and used without further purification. Butvar B-98 and PDMS-OH 36,000 and 18,000 were obtained from Acros Organics www.acros.com (accessed on 10 November 2021) (part of Thermo Fisher Ltd., Scientific) Geel, Belgium, and Fluorochem, Glossop, UK, respectively. PDMS-OH 4200 and ~550 were obtained from VWR International, Lutterworth, UK, and Merck Ltd., Darmstadt, Germany.

### 5.2. Analytical Ultracentrifugation

A Beckman Optima XL-I analytical ultracentrifuge with Rayleigh interference optics was used at 20.0 °C. Furthermore, 12 mm optical path length double sector cells with titanium centrepieces were employed. A description of the sedimentation velocity and sedimentation equilibrium techniques are given in Harding et al. [32], Dam and Schuck, [20] Schuck et al. [22], and Harding et al. [33].

#### 5.2.1. Sedimentation Velocity

An amount of 405 μL of the previous loading concentrations (0.5 to 4.0 mg/mL) of polymer (Butvar B-98 or PDMS-OH) in isopropanol were added to each of the cells. A rotational speed of 49,000 rpm was used, and the samples were centrifuged overnight. The weighted average sedimentation coefficient and the distributions of sedimentation coefficient c(s) vs. s were obtained by analysis with the SEDFIT procedure [20].

#### 5.2.2. Sedimentation Equilibrium

Loading concentrations of 0.5 to 4.0 mg/mL of polymer in isopropanol were used. An amount of 100 μL of each concentration was injected into the sample solution channel of the cell. Isopropanol was used as the reference solvent. For Butvar B-98, the experiment was carried out at a rotational speed of 22,000 rpm over 2 days. For PS36000 and PS18000, the experiments were carried out over 2 days using rotational speeds of 22,000 rpm and 30,000 rpm, respectively. The experiments for PS4200 and PS550 were both carried out at a speed of 49,000 rpm and left overnight. The results were analysed with SEDFIT-MSTAR [22] in order to obtain the apparent weight-average molar mass *M*_w,app_, making use of the M* extrapolation [34] and the hinge point method [21]. No significant concentration dependence was observed, suggesting that non-ideality was not significant. The data obtained from the highest concentration (4.0 mg/mL) was additionally analysed with the MULTISIG algorithm [23] to evaluate the molar mass distribution.

### 5.3. Viscosity Measurements

An Anton Paar AMVn (Graz, Austria) rolling ball viscometer was used at a temperature of 10.0 °C. Its closed capillary system is more suitable for working with volatile solvent systems compared to conventional Ostwald viscometers. The intrinsic viscosity measurements were carried out using a 6.0 mg/mL concentration of Butvar B-98 and PDMS-OH in isopropanol and turpentine. The intrinsic viscosity was then calculated with the Solomon–Ciuta Equation (1).

### 5.4. Infusion Experiment

The sample of Oseberg wood (Figure 2 was injected with a concentrated suspension (~5% or 50 g/L) of PS36000 solution in siloxane in turpentine. The fragment was injected for a period of 11 days and then left to dry for 34 days. The retreatment was conducted in a fume hood at room temperature.

## Figures and Tables

**Table 1:** Molar mass values for PDMS-OH siloxanes in isopropanol *M*_w_: weight-average molar mass values in isopropanol from sedimentation equilibrium in the analytical ultracentrifuge. *M*_r_: relative molar mass values relative to polystyrene standards.

Polymer	*M*_r_ (Da)	*M*_w_ (Da)
PS36000	36,000	52,500 ± 3000
PS18000	18,000	38,800 ± 1500
PS4200	4200	6200 ± 700
PS550	~550	1550 ± 50

**Table 2:** A comparison of the $\left[\eta \right]$ values obtained for all the tested polymers.

	$\left[\eta \right]\;\mathrm{in}\;\mathrm{Isopropanol}\;(\mathrm{mL}/g)$	$\left[\eta \right]\;\mathrm{in}\;\mathrm{Turpentine}\;(\mathrm{mL}/g)$
Butvar B-98	57± 3	N/A
Siloxane PS36000	6.0 ± 0.3	22.4 ± 1.1
Siloxane PS18000	7.5 ± 0.4	15.8 ± 0.8
Siloxane PS4200	4.1 ± 0.2	5.5 ± 0.3
Siloxane PS550	3.7 ± 0.2	*

* flow time increment too small to record.

**Table 3:** Viscosity increment ν for different degrees of solvent association (dynamic binding) and corresponding axial ratios (*a*/*b*) for Butvar B-98 and PDMS-OH polymers in isopropanol.

		$\mathrm{Degree}\;\mathrm{of}\;\mathrm{Solvent}\;\mathrm{Association}\;\left(v_{s}/\overset{¯}{v}\right)$		
		1	1.2	1.4
Butvar B-98	ν	64 ± 3	53 ± 3	45 ± 3
	(a/b)	27	24	22
PS36000	ν	5.9 ± 2.0	4.9 ± 2.0	4.2 ± 2.0
	(a/b)	5	4	4
PS18000	ν	7.2 ± 2.3	6.0 ± 2.3	5.2 ± 2.3
	(a/b)	6	5	5
PS4200	ν	4.0 ± 0.7	3.3 ± 0.7	2.8 ± 0.7
	(a/b)	3	3	3
PS550	ν	3.6 ± 0.2	3.0 ± 0.2	2.5 ± 0.2
	(a/b)	3	2	1

**Table 4:** Viscosity increment ν for PDMS-OH polymers in turpentine.

		$\mathrm{Degree}\;\mathrm{of}\;\mathrm{Solvent}\;\mathrm{Association}\;\left(v_{s}/\overset{¯}{v}\right)$		
		1	1.2	1.4
PS36000	ν	22.0	18.3	15.7
	(a/b)	14	12	11
PS18000	ν	15	13	11
	(a/b)	11	10	9
PS4200	ν	5.4	4.5	3.8
	(a/b)	5	4	3

## Supplementary Materials

The following are available online at https://www.mdpi.com/article/10.3390/molecules27072133/s1, Table S1. Apparent weight-average molar masses *M*_w,app_ and polydispersity Đ values obtained from sedimentation equilibrium of Butvar B-98; Table S2. Apparent weight-average molar masses *M*_w,app_ and polydispersity Đ values for PS36000 in isopropanol at different concentrations; Table S3. Apparent weight-average molar masses *M*_w,app_ and polydispersity Đ values for PS18000 in isopropanol at different concentrations; Table S4. Apparent weight-average molar masses *M*_w,app_ and polydispersity Đ values for PS4200 in isopropanol at different concentrations; Table S5. Apparent weight-average molar masses *M*_w,app_ and polydispersity Đ values for PS550 in isopropanol at different concentrations; Figure S1. Conformations (equivalent hydrodynamic ellipsoids) for ν at $\left(v_{s}/\bar{v}\right)$ = 1.2 of the polymers, using the programme ELLIPS1. (a) = PS18000 in isopropanol; (b) = PS4200 in isopropanol; (c) = PS550 in isopropanol; (d) P36000 in turpentine; (e) = PS18000 in turpentine; (f) = P4200 in turpentine.

## Sample Availability

Samples of the polymers are commercially available.

## Abbreviations

GPC gel-permeation chromatography; *M*_n_, *M*_w_, *M*_z_ number, weight, z-average molar masses or molecular weights; *M*_app_ weight-average molar mass; PDMS-OH hydroxy-terminated polydimethylsiloxane; S Svedberg units; *s* sedimentation coefficient; *s*_20,w_ sedimentation coefficient normalised to standard solvent conditions (density & viscosity of water at 20.0 °C); $\bar{v}$ partial specific volume; *v_s_* swollen specific volume; [*η*] intrinsic viscosity; ν viscosity increment.
