# Supplementary material for: Comparative Hydrodynamic Study on Non-Aqueous Soluble Archaeological Wood Consolidants: Butvar B-98 and PDMS-OH Siloxanes
Source: Molecules. 2022 Mar 25;27(7):2133. doi: 10.3390/molecules27072133 (PMC9000765; doi:10.3390/molecules27072133)
Supplement: Supplementary file 1 [file molecules-27-02133-s001.zip › molecules-1625715-supplementary.pdf]

Supplementary Materials

Table S1. Apparent weight average molar masses $M_{w,app}$ and polydispersity $Đ$ values obtained from sedimentation equilibrium of Butvar B-98.

Concentration (mg/ml)	$M_{w,app}$ (kDa)	$Đ$ ($M_{z,app}/M_{w,app}$)
0.5	51.5	2.7
0.75	39.2	2.4
1.0	50.9	1.8
1.5	50.2	2.1
2.0	43.8	1.8
3.0	41.1	1.7
4.0	39.4	1.6

Table S2. Apparent weight average molar masses $M_{w,app}$ and polydispersity $Đ$ values for PS36000 in isopropanol at different concentrations.

Concentration (mg/mL)	$^{a}M_{w,app}$ (kDa)	$^{b}M_{w,app}$ (kDa)	$Đ$ ($M_{z,app}/M_{w,app}$)
0.5	48.7	44.4	1.0
1.0	49.0	38.2	1.3
2.0	35.6	34.4	1.6
3.0	29.1	28.9	1.0
4.0	27.7	26.8	1.3

a: M* method. b: hinge point method.

Table S3. Apparent weight average molar masses $M_{w,app}$ and polydispersity $Đ$ values for PS18000 in isopropanol at different concentrations.

Concentration (mg/mL)	$^{a}M_{w,app}$ (kDa)	$^{b}M_{w,app}$ (kDa)	$Đ$ ($M_{z,app}/M_{w,app}$)
1.0	33.6	31.0	1.3
2.0	29.8	25.2	1.3
3.0	22.9	18.5	1.3
4.0	22.3	19.9	1.4

a: M* method. b: hinge point method.

Table S4. Apparent weight average molar masses $M_{w,app}$ and polydispersity $Đ$ values for PS4200 in isopropanol at different concentrations.

Concentration (mg/mL)	$^{a}M_{w,app}$ (kDa)	$^{b}M_{w,app}$ (kDa)	$Đ$ ($M_{z}/M_{w,app}$)
1.0	5.9	5.2	3.8
1.5	4.6	3.8	5.4
2.0	5.7	5.1	2.9
3.0	5.1	4.5	2.7
4.0	3.9	3.4	5.1

a: M* method. b: hinge point method.

Table S5. Apparent weight average molar masses $M_{w,app}$ (M^* method) and polydispersity $Đ$ values for PS550 in isopropanol.

Concentration (mg/mL)	$M_{w,app}$ (kDa)	$Đ$ ($M_{z}/M_{w,app}$)
3.0	1.6	1.8
4.0	1.5	1.8

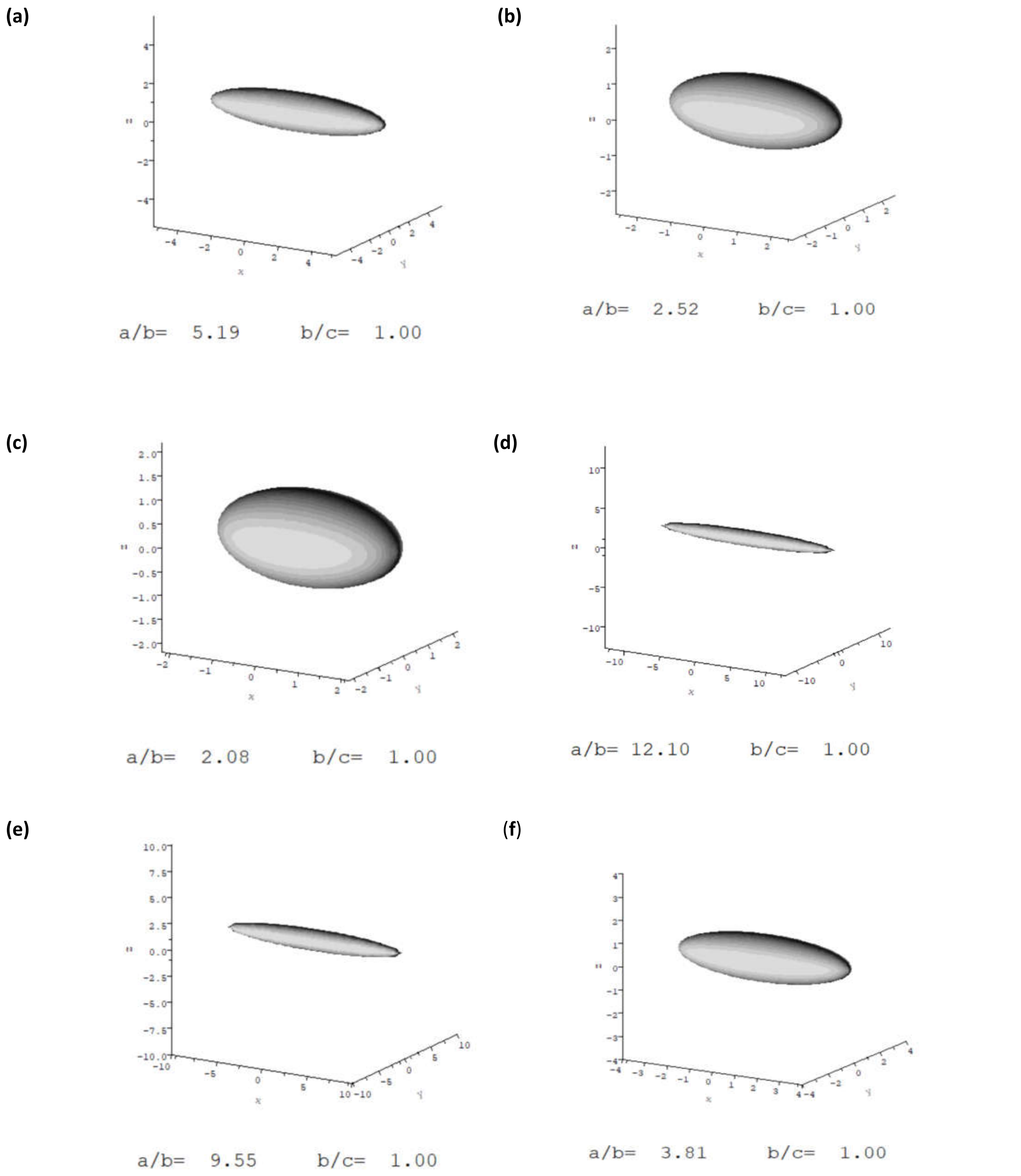

Figure S1. Conformations (equivalent hydrodynamic ellipsoids) for ν at $(v_s/\bar{v}) = 1.2$ using the programme ELLIPS1. (a) = PS18000 in isopropanol; (b) = PS4200 in isopropanol; (c) = PS550 in isopropanol; (d) P36000 in turpentine; (e) = PS18000 in turpentine; (f) = P4200 in turpentine.
